# Octa­rubidium di-μ-sulfato-κ^4^ *O*:*O*′-bis­[*cis*-dioxido-*cis*-disulfatotungstate(VI)]

**DOI:** 10.1107/S1600536809046431

**Published:** 2009-11-11

**Authors:** Kenny Ståhl, Rolf W. Berg

**Affiliations:** a Technical University of Denmark, Department of Chemistry, Building 207, DK-2800 Lyngby, Denmark

## Abstract

The title compound, Rb_8_[W_2_O_4_(SO_4_)_6_], was precipitated from a melt of tungsten(VI) oxide and rubidium sulfate in rubidium disulfate. The unit cell contains two discrete [{W^VI^O_2_(SO_4_)_2_}_2_(μ-SO_4_)_2_]^8−^ units connected by Rb–O coord­ination. The W atom is octahedrally surrounded by two oxide ligands, two terminal sulfate ligands and two bridging sulfate groups. One Rb atom is coordinated by eight O atoms, whereas the three other Rb atoms are coordinated by nine O atoms from sulfate and oxide groups, leading to distorted [RbO_*x*_] polyhedra.

## Related literature

For methods used in the synthesis, see: Berg *et al.* (2006 ▶); Borup *et al.* (1990 ▶); Nørbygaard *et al.* (1998 ▶). For the crystal structure of the potassium analog, see: Schäffer & Berg (2005 ▶).
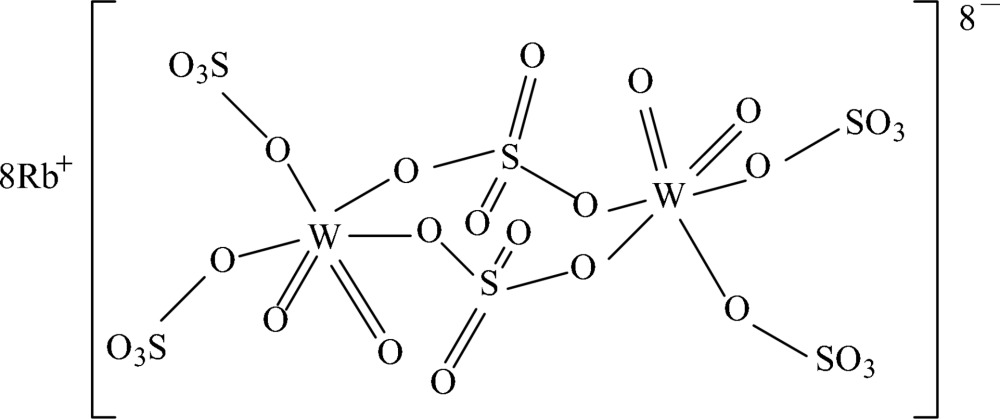


## Experimental

### 

#### Crystal data

Rb_8_[W_2_O_4_(SO_4_)_6_]
*M* *_r_* = 1691.86
Monoclinic, 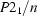

*a* = 9.6405 (5) Å
*b* = 13.9890 (7) Å
*c* = 10.7692 (5) Å
β = 90.472 (1)°
*V* = 1452.30 (12) Å^3^
*Z* = 2
Mo *K*α radiation
μ = 21.77 mm^−1^
*T* = 120 K
0.45 × 0.20 × 0.05 mm

#### Data collection

Bruker SMART APEX diffractometer
Absorption correction: multi-scan (*SADABS*; Sheldrick, 2002 ▶) *T*_min_ = 0.077, *T*_max_ = 0.59
18895 measured reflections
3496 independent reflections
3360 reflections with *I* > 2σ(*I*)
*R*_int_ = 0.036

#### Refinement

*R*[*F* ^2^ > 2σ(*F* ^2^)] = 0.020
*wR*(*F* ^2^) = 0.052
*S* = 1.11
3496 reflections
200 parameters
Δρ_max_ = 1.35 e Å^−3^
Δρ_min_ = −1.51 e Å^−3^

### 

Data collection: *SMART* (Bruker, 2002 ▶); cell refinement: *SAINT-Plus* (Bruker, 2002 ▶); data reduction: *SAINT-Plus*; program(s) used to solve structure: *SHELXTL* (Sheldrick, 2008 ▶); program(s) used to refine structure: *SHELXTL*; molecular graphics: *ORTEP-3* (Farrugia, 1997 ▶) and *ATOMS* (Dowty, 2000 ▶); software used to prepare material for publication: *SHELXTL*.

## Supplementary Material

Crystal structure: contains datablocks I, global. DOI: 10.1107/S1600536809046431/fi2090sup1.cif

Structure factors: contains datablocks I. DOI: 10.1107/S1600536809046431/fi2090Isup2.hkl

Additional supplementary materials:  crystallographic information; 3D view; checkCIF report

## Figures and Tables

**Table 1:** Selected bond lengths (Å)

W1—O1	1.716 (2)
W1—O2	1.721 (2)
W1—O3	1.960 (2)
W1—O4	2.009 (2)
W1—O5	2.097 (2)
W1—O6	2.254 (2)
Rb1—O1	2.877 (2)
Rb1—O8	2.931 (2)
Rb1—O7	2.939 (3)
Rb1—O9^i^	2.955 (3)
Rb1—O6^ii^	3.009 (2)
Rb1—O11^iii^	3.010 (2)
Rb1—O2^iii^	3.084 (2)
Rb1—O5^ii^	3.185 (2)
Rb2—O14^iv^	2.761 (3)
Rb2—O7^v^	2.893 (2)
Rb2—O9^vi^	2.903 (3)
Rb2—O2	2.939 (3)
Rb2—O13^ii^	2.986 (2)
Rb2—O8	3.082 (3)
Rb2—O10^v^	3.106 (3)
Rb2—O9	3.337 (3)
Rb2—O1	3.374 (3)
Rb3—O11	2.776 (2)
Rb3—O10^ii^	2.788 (3)
Rb3—O7^v^	2.874 (2)
Rb3—O14^vii^	2.935 (2)
Rb3—O12^i^	3.101 (2)
Rb3—O11^vii^	3.170 (2)
Rb3—O12^v^	3.173 (3)
Rb3—O1	3.220 (2)
Rb3—O13^v^	3.225 (3)
Rb4—O8^viii^	2.910 (3)
Rb4—O3^i^	2.914 (2)
Rb4—O10	2.941 (3)
Rb4—O14	2.964 (2)
Rb4—O13^i^	2.981 (3)
Rb4—O12^i^	3.103 (3)
Rb4—O6^i^	3.221 (2)
Rb4—O5	3.253 (2)
Rb4—O4	3.423 (2)
S1—O11	1.445 (2)
S1—O14	1.449 (2)
S1—O6^ix^	1.490 (2)
S1—O4	1.531 (2)
S2—O12	1.451 (3)
S2—O9	1.452 (3)
S2—O8	1.454 (3)
S2—O3	1.575 (2)
S3—O13	1.461 (2)
S3—O7	1.464 (3)
S3—O10	1.464 (3)
S3—O5	1.528 (2)

Symmetry codes: (i) 
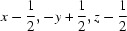
; (ii) 
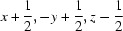
; (iii) 
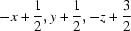
; (iv) 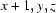; (v) 
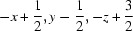
; (vi) 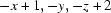; (vii) 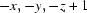; (viii) 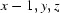; (ix) 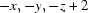.

**Fig. 1.**
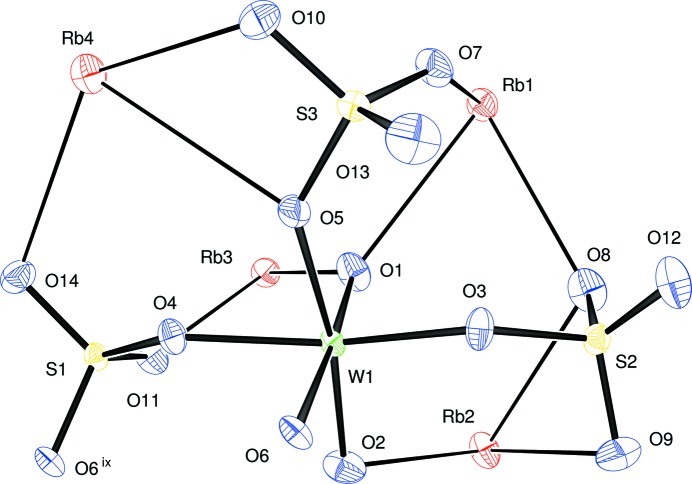
The asymmetric unit of (I) showing 50% probability displacement ellipsoids and the atomic numbering. O6iv was added to complete the S1 coordination.

**Fig. 2.**
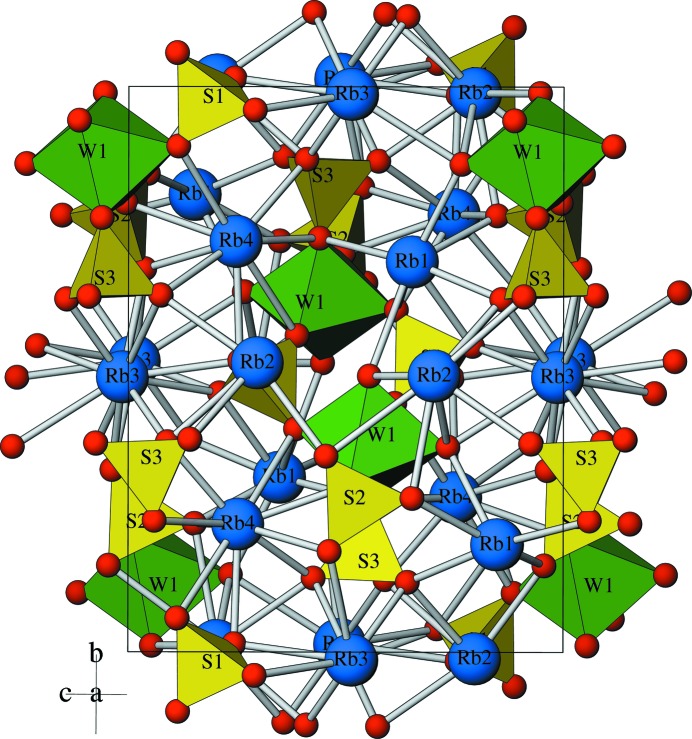
The crystal packing of (I) viewed down the a axis. W atoms are in the centers of the octahedra and S atoms are in the centers of the tetrahedra. Rb are shown as large and O as small spheres.

## supplementary crystallographic information

### Comment

Tungsten trioxide is known as a highly inert solid, practically insoluble in acids. It was discovered that WO_3_ can be dissolved in considerable amounts in acidic sulfate melts at high temperatures (Schäffer and Berg, 2005 and Berg *et al.*, 2006). When therefore WO_3_, Rb_2_SO_4_ and Rb_2_S_2_O_7_ are mixed in varying molar amounts in sealed ampoules and heated for equilibration in a rocking furnace at appr. 600 °C for appr. 1 hr, clear melts are formed (Rb_2_S_2_O_7_ is hygroscopic and needs to be handled in a dry box). It has been shown that [WO_2_]^2+^ ions formed are solvated by SO_4_^2-^ ions. The stoichiometry of the reaction have been determined fairly accurately as 1:1:1, or 2WO_3_ + 2*M*_2_SO_4_ + 2*M*_2_S_2_O_7_→*M*_8_[(WO_2_)_2_(µ-SO_4_)_2_(SO_4_)_4_] for the case of *M* = K, Schäffer and Berg, 2005. Here we have studied the case of *M* = Rb and have found closely analogous results for single crystals of the compound, 1WO_3_:1Rb_2_S_2_O_7_:1Rb_2_SO_4_. The main difference for *M* = Rb as compared to *M* = K is as expected in the *M* - O coordination: Rb1/K1, CN = 8/8, <*M* - O> = 2.999 (1)/2.822 (1); Rb2/K2, CN = 9/7, <*M* - O> = 3.042 (1)/2.825 (1); Rb3/K3, CN = 9/8, <*M* - O> = 3.029 (1)/2.909 (1); Rb4/K4, CN = 9/9, <*M* - O> = 3.079 (1)/2.945 (1). As the oxo-sulfatotungstate groups are extended in the b-direction, the major changes in the unit cell axes are observered in the a- and c- directions.

### Experimental

The crystals were grown from a melt of 20.6 mol % tungsten trioxide and 19.9 mol % rubidium sulfate in rubidium disulfate, using the methods described by Borup *et al.* (1990), Nørbygaard *et al.* (1998) and Berg *et al.* (2006).

### Crystal data

**Table:**

Rb_8_[W_2_O_4_(SO_4_)_6_]	*F*(000) = 1528
*M**_r_* = 1691.86	*D*_x_ = 3.869 Mg m^−^^3^
Monoclinic, *P*2_1_/*n*	Mo *K*α radiation, λ = 0.71073 Å
Hall symbol: -P 2yn	Cell parameters from 6979 reflections
*a* = 9.6405 (5) Å	θ = 2.4–28.0°
*b* = 13.9890 (7) Å	µ = 21.77 mm^−^^1^
*c* = 10.7692 (5) Å	*T* = 120 K
β = 90.472 (1)°	Irregular, colourless
*V* = 1452.30 (12) Å^3^	0.45 × 0.20 × 0.05 mm
*Z* = 2	

### Data collection

**Table:**

Bruker SMART APEX diffractometer	3496 independent reflections
Radiation source: fine-focus sealed tube	3360 reflections with *I* > 2σ(*I*)
graphite	*R*_int_ = 0.036
ω scan, frame data integration	θ_max_ = 28.0°, θ_min_ = 2.4°
Absorption correction: multi-scan (*SADABS*; Sheldrick, 2002)	*h* = −12→12
*T*_min_ = 0.077, *T*_max_ = 0.59	*k* = −18→18
18895 measured reflections	*l* = −14→14

### Refinement

**Table:**

Refinement on *F*^2^	Primary atom site location: structure-invariant direct methods
Least-squares matrix: full	Secondary atom site location: difference Fourier map
*R*[*F*^2^ > 2σ(*F*^2^)] = 0.020	*w* = 1/[σ^2^(*F*_o_^2^) + (0.0287*P*)^2^ + 1.1918*P*] where *P* = (*F*_o_^2^ + 2*F*_c_^2^)/3
*wR*(*F*^2^) = 0.052	(Δ/σ)_max_ = 0.001
*S* = 1.11	Δρ_max_ = 1.35 e Å^−^^3^
3496 reflections	Δρ_min_ = −1.51 e Å^−^^3^
200 parameters	Extinction correction: *SHELXTL* (Sheldrick, 2008), Fc^*^=kFc[1+0.001xFc^2^λ^3^/sin(2θ)]^-1/4^
0 restraints	Extinction coefficient: 0.00175 (10)

### Special details

**Table:**

Experimental. Oxford Cryosystem liquid nitrogen cryostream cooler
Geometry. All e.s.d.'s are estimated using the full covariance matrix. The cell e.s.d.'s are taken into account individually in the estimation of e.s.d.'s in distances, angles and torsion angles; correlations between e.s.d.'s in cell parameters are only used when they are defined by crystal symmetry.
Refinement. A series of identical frames was collected twice during the experiment to monitor decay. No decay was detected and decay correction was not applied. Systematic conditions suggested the unambiguous space group. The structure was solved by direct methods (Sheldrick, 2008). The space group choice was confirmed by successful convergence of the full-matrix least-squares refinement on *F*^2^ (Sheldrick, 2008). Refinement of *F*^2^ against ALL reflections. The weighted *R*-factor *wR* and goodness of fit *S* are based on *F*^2^, conventional *R*-factors *R* are based on *F*, with *F* set to zero for negative *F*^2^. The threshold expression of *F*^2^ > 2σ(*F*^2^) is used only for calculating *R*-factors(gt) *etc*. and is not relevant to the choice of reflections for refinement. *R*-factors based on *F*^2^ are statistically about twice as large as those based on *F*, and *R*- factors based on ALL data will be even larger. The highest peak in the final difference Fourier map was 0.85Å and the deepest hole was 0.78Å from W1. The final map had no other significant features. A final analysis of variance between observed and calculated structure factors showed no dependence on amplitude or resolution.

### Fractional atomic coordinates and isotropic or equivalent isotropic displacement parameters (Å^2^)

**Table:**

	*x*	*y*	*z*	*U*_iso_*/*U*_eq_	
W1	0.168190 (12)	0.117132 (8)	0.917586 (11)	0.00604 (6)	
Rb1	0.31202 (3)	0.30953 (2)	0.65221 (3)	0.01095 (8)	
Rb2	0.50882 (3)	0.01145 (2)	0.79641 (3)	0.01149 (8)	
Rb3	0.19055 (3)	0.01388 (2)	0.51347 (3)	0.01057 (8)	
Rb4	−0.23085 (3)	0.22589 (2)	0.74755 (3)	0.01173 (8)	
S1	−0.07712 (8)	−0.01972 (5)	0.80402 (7)	0.00671 (15)	
S2	0.46037 (8)	0.22894 (6)	0.97953 (7)	0.00823 (15)	
S3	0.03580 (8)	0.34141 (5)	0.94629 (7)	0.00726 (15)	
O1	0.2116 (3)	0.13695 (17)	0.7656 (2)	0.0126 (5)	
O2	0.2550 (3)	0.01169 (17)	0.9456 (2)	0.0134 (5)	
O3	0.3015 (2)	0.20560 (17)	0.9939 (2)	0.0105 (5)	
O4	−0.0203 (2)	0.06004 (16)	0.8874 (2)	0.0095 (4)	
O5	0.0314 (2)	0.23235 (16)	0.9395 (2)	0.0113 (5)	
O6	0.0953 (2)	0.10525 (15)	1.1153 (2)	0.0086 (4)	
O7	0.1394 (3)	0.37617 (16)	0.8585 (2)	0.0123 (5)	
O8	0.4896 (3)	0.22782 (18)	0.8473 (2)	0.0146 (5)	
O9	0.5342 (3)	0.1533 (2)	1.0446 (3)	0.0221 (6)	
O10	−0.1042 (3)	0.37142 (17)	0.9097 (2)	0.0143 (5)	
O11	0.0230 (3)	−0.04158 (17)	0.7090 (2)	0.0118 (5)	
O12	0.4761 (3)	0.32204 (18)	1.0374 (2)	0.0160 (5)	
O13	0.0695 (3)	0.36869 (18)	1.0740 (2)	0.0168 (5)	
O14	−0.2088 (3)	0.01462 (17)	0.7559 (2)	0.0121 (5)	

### Atomic displacement parameters (Å^2^)

**Table:**

	*U*^11^	*U*^22^	*U*^33^	*U*^12^	*U*^13^	*U*^23^
W1	0.00608 (8)	0.00633 (8)	0.00575 (8)	−0.00051 (4)	0.00225 (5)	−0.00028 (4)
Rb1	0.01021 (15)	0.01196 (15)	0.01072 (15)	−0.00113 (11)	0.00177 (11)	0.00109 (11)
Rb2	0.01089 (16)	0.01125 (15)	0.01242 (16)	−0.00146 (11)	0.00470 (11)	−0.00220 (11)
Rb3	0.01010 (15)	0.01395 (15)	0.00769 (15)	−0.00045 (11)	0.00251 (11)	0.00227 (10)
Rb4	0.01259 (16)	0.01381 (15)	0.00883 (15)	−0.00152 (12)	0.00221 (11)	−0.00042 (11)
S1	0.0069 (3)	0.0080 (3)	0.0052 (3)	−0.0015 (3)	0.0008 (3)	0.0002 (3)
S2	0.0065 (3)	0.0102 (4)	0.0080 (4)	−0.0011 (3)	0.0004 (3)	0.0005 (3)
S3	0.0086 (4)	0.0070 (3)	0.0062 (3)	0.0008 (3)	0.0016 (3)	−0.0007 (3)
O1	0.0157 (12)	0.0143 (11)	0.0077 (11)	−0.0046 (10)	0.0025 (9)	−0.0001 (9)
O2	0.0144 (12)	0.0098 (11)	0.0161 (12)	0.0047 (9)	0.0057 (9)	0.0011 (9)
O3	0.0071 (11)	0.0141 (11)	0.0104 (11)	−0.0017 (9)	0.0015 (8)	−0.0033 (9)
O4	0.0099 (11)	0.0093 (10)	0.0094 (11)	−0.0024 (9)	0.0007 (8)	−0.0019 (8)
O5	0.0088 (11)	0.0081 (10)	0.0170 (12)	0.0002 (9)	0.0006 (9)	0.0009 (9)
O6	0.0110 (11)	0.0068 (10)	0.0080 (11)	−0.0025 (8)	0.0025 (9)	0.0006 (8)
O7	0.0123 (12)	0.0127 (12)	0.0120 (12)	−0.0024 (9)	0.0043 (9)	0.0030 (8)
O8	0.0156 (12)	0.0178 (12)	0.0105 (12)	−0.0028 (10)	0.0057 (9)	−0.0012 (9)
O9	0.0171 (13)	0.0202 (14)	0.0288 (15)	0.0015 (11)	−0.0074 (11)	0.0102 (11)
O10	0.0099 (12)	0.0123 (11)	0.0207 (14)	0.0039 (9)	0.0018 (10)	−0.0009 (9)
O11	0.0142 (12)	0.0130 (11)	0.0084 (11)	−0.0017 (9)	0.0049 (8)	−0.0008 (9)
O12	0.0153 (12)	0.0157 (12)	0.0170 (13)	−0.0054 (10)	0.0020 (10)	−0.0047 (10)
O13	0.0250 (15)	0.0173 (12)	0.0079 (12)	0.0004 (11)	−0.0014 (10)	−0.0039 (9)
O14	0.0110 (12)	0.0151 (12)	0.0102 (11)	0.0011 (9)	−0.0017 (9)	0.0014 (9)

### Geometric parameters (Å, °)

**Table:**

W1—O1	1.716 (2)	Rb3—O12^i^	3.101 (2)
W1—O2	1.721 (2)	Rb3—O11^vii^	3.170 (2)
W1—O3	1.960 (2)	Rb3—O12^v^	3.173 (3)
W1—O4	2.009 (2)	Rb3—O1	3.220 (2)
W1—O5	2.097 (2)	Rb3—O13^v^	3.225 (3)
W1—O6	2.254 (2)	Rb4—O8^viii^	2.910 (3)
Rb1—O1	2.877 (2)	Rb4—O3^i^	2.914 (2)
Rb1—O8	2.931 (2)	Rb4—O10	2.941 (3)
Rb1—O7	2.939 (3)	Rb4—O14	2.964 (2)
Rb1—O9^i^	2.955 (3)	Rb4—O13^i^	2.981 (3)
Rb1—O6^ii^	3.009 (2)	Rb4—O12^i^	3.103 (3)
Rb1—O11^iii^	3.010 (2)	Rb4—O6^i^	3.221 (2)
Rb1—O2^iii^	3.084 (2)	Rb4—O5	3.253 (2)
Rb1—O5^ii^	3.185 (2)	Rb4—O4	3.423 (2)
Rb2—O14^iv^	2.761 (3)	S1—O11	1.445 (2)
Rb2—O7^v^	2.893 (2)	S1—O14	1.449 (2)
Rb2—O9^vi^	2.903 (3)	S1—O6^ix^	1.490 (2)
Rb2—O2	2.939 (3)	S1—O4	1.531 (2)
Rb2—O13^ii^	2.986 (2)	S2—O12	1.451 (3)
Rb2—O8	3.082 (3)	S2—O9	1.452 (3)
Rb2—O10^v^	3.106 (3)	S2—O8	1.454 (3)
Rb2—O9	3.337 (3)	S2—O3	1.575 (2)
Rb2—O1	3.374 (3)	S3—O13	1.461 (2)
Rb3—O11	2.776 (2)	S3—O7	1.464 (3)
Rb3—O10^ii^	2.788 (3)	S3—O10	1.464 (3)
Rb3—O7^v^	2.874 (2)	S3—O5	1.528 (2)
Rb3—O14^vii^	2.935 (2)		
			
O1—W1—O2	100.59 (12)	O7^v^—Rb3—O12^i^	146.54 (7)
O1—W1—O3	97.67 (11)	O14^vii^—Rb3—O12^i^	103.19 (7)
O2—W1—O3	98.71 (11)	O11—Rb3—O11^vii^	103.26 (6)
O1—W1—O4	97.90 (10)	O10^ii^—Rb3—O11^vii^	94.90 (7)
O2—W1—O4	97.20 (11)	O7^v^—Rb3—O11^vii^	143.97 (6)
O3—W1—O4	155.24 (9)	O14^vii^—Rb3—O11^vii^	46.63 (6)
O1—W1—O5	98.15 (11)	O12^i^—Rb3—O11^vii^	62.82 (6)
O2—W1—O5	160.75 (11)	O11—Rb3—O12^v^	66.26 (7)
O3—W1—O5	83.02 (9)	O10^ii^—Rb3—O12^v^	141.04 (7)
O4—W1—O5	75.81 (9)	O7^v^—Rb3—O12^v^	78.58 (6)
O1—W1—O6	173.62 (10)	O14^vii^—Rb3—O12^v^	75.47 (7)
O2—W1—O6	85.75 (10)	O12^i^—Rb3—O12^v^	107.66 (6)
O3—W1—O6	81.90 (9)	O11^vii^—Rb3—O12^v^	69.40 (6)
O4—W1—O6	80.50 (9)	O11—Rb3—O1	62.79 (7)
O5—W1—O6	75.48 (9)	O10^ii^—Rb3—O1	89.42 (7)
O1—Rb1—O8	64.22 (7)	O7^v^—Rb3—O1	85.54 (6)
O1—Rb1—O7	75.57 (7)	O14^vii^—Rb3—O1	154.44 (7)
O8—Rb1—O7	84.99 (7)	O12^i^—Rb3—O1	64.64 (6)
O1—Rb1—O9^i^	90.40 (8)	O11^vii^—Rb3—O1	127.46 (6)
O8—Rb1—O9^i^	150.64 (8)	O12^v^—Rb3—O1	128.76 (6)
O7—Rb1—O9^i^	73.96 (7)	O11—Rb3—O13^v^	117.97 (7)
O1—Rb1—O6^ii^	134.22 (7)	O10^ii^—Rb3—O13^v^	74.48 (7)
O8—Rb1—O6^ii^	73.97 (7)	O7^v^—Rb3—O13^v^	46.39 (7)
O7—Rb1—O6^ii^	119.62 (6)	O14^vii^—Rb3—O13^v^	64.96 (6)
O9^i^—Rb1—O6^ii^	134.35 (7)	O12^i^—Rb3—O13^v^	166.10 (7)
O1—Rb1—O11^iii^	123.25 (6)	O11^vii^—Rb3—O13^v^	108.61 (6)
O8—Rb1—O11^iii^	67.10 (7)	O12^v^—Rb3—O13^v^	77.31 (7)
O7—Rb1—O11^iii^	72.87 (7)	O1—Rb3—O13^v^	122.86 (6)
O9^i^—Rb1—O11^iii^	123.13 (7)	O8^viii^—Rb4—O3^i^	116.73 (7)
O6^ii^—Rb1—O11^iii^	46.76 (6)	O8^viii^—Rb4—O10	98.95 (7)
O1—Rb1—O2^iii^	147.59 (7)	O3^i^—Rb4—O10	106.38 (7)
O8—Rb1—O2^iii^	136.20 (7)	O8^viii^—Rb4—O14	93.69 (7)
O7—Rb1—O2^iii^	81.33 (6)	O3^i^—Rb4—O14	110.37 (7)
O9^i^—Rb1—O2^iii^	61.21 (7)	O10—Rb4—O14	130.00 (7)
O6^ii^—Rb1—O2^iii^	77.25 (6)	O8^viii^—Rb4—O13^i^	68.94 (7)
O11^iii^—Rb1—O2^iii^	69.12 (6)	O3^i^—Rb4—O13^i^	68.39 (7)
O1—Rb1—O5^ii^	112.24 (7)	O10—Rb4—O13^i^	160.41 (7)
O8—Rb1—O5^ii^	93.25 (6)	O14—Rb4—O13^i^	67.81 (7)
O7—Rb1—O5^ii^	170.30 (6)	O8^viii^—Rb4—O12^i^	150.99 (7)
O9^i^—Rb1—O5^ii^	110.80 (7)	O3^i^—Rb4—O12^i^	46.59 (6)
O6^ii^—Rb1—O5^ii^	50.90 (6)	O10—Rb4—O12^i^	108.45 (7)
O11^iii^—Rb1—O5^ii^	97.65 (6)	O14—Rb4—O12^i^	76.14 (7)
O2^iii^—Rb1—O5^ii^	93.51 (6)	O13^i^—Rb4—O12^i^	82.09 (7)
O14^iv^—Rb2—O7^v^	113.73 (7)	O8^viii^—Rb4—O6^i^	71.11 (7)
O14^iv^—Rb2—O9^vi^	104.59 (7)	O3^i^—Rb4—O6^i^	53.40 (6)
O7^v^—Rb2—O9^vi^	75.43 (7)	O10—Rb4—O6^i^	88.10 (6)
O14^iv^—Rb2—O2	155.92 (7)	O14—Rb4—O6^i^	141.38 (6)
O7^v^—Rb2—O2	84.64 (7)	O13^i^—Rb4—O6^i^	73.59 (6)
O9^vi^—Rb2—O2	63.56 (7)	O12^i^—Rb4—O6^i^	99.87 (6)
O14^iv^—Rb2—O13^ii^	70.38 (7)	O8^viii^—Rb4—O5	118.84 (7)
O7^v^—Rb2—O13^ii^	90.22 (7)	O3^i^—Rb4—O5	119.85 (6)
O9^vi^—Rb2—O13^ii^	161.61 (8)	O10—Rb4—O5	44.51 (6)
O2—Rb2—O13^ii^	127.42 (7)	O14—Rb4—O5	87.33 (6)
O14^iv^—Rb2—O8	94.19 (7)	O13^i^—Rb4—O5	154.80 (6)
O7^v^—Rb2—O8	135.64 (7)	O12^i^—Rb4—O5	88.19 (6)
O9^vi^—Rb2—O8	131.74 (8)	O6^i^—Rb4—O5	131.24 (6)
O2—Rb2—O8	81.38 (7)	O8^viii^—Rb4—O4	113.04 (6)
O13^ii^—Rb2—O8	66.65 (7)	O3^i^—Rb4—O4	124.58 (6)
O14^iv^—Rb2—O10^v^	66.37 (7)	O10—Rb4—O4	88.06 (6)
O7^v^—Rb2—O10^v^	47.37 (7)	O14—Rb4—O4	43.02 (6)
O9^vi^—Rb2—O10^v^	88.01 (8)	O13^i^—Rb4—O4	110.63 (6)
O2—Rb2—O10^v^	130.12 (7)	O12^i^—Rb4—O4	77.99 (6)
O13^ii^—Rb2—O10^v^	73.72 (7)	O6^i^—Rb4—O4	174.76 (6)
O8—Rb2—O10^v^	139.93 (7)	O5—Rb4—O4	44.32 (5)
O14^iv^—Rb2—O9	92.92 (7)	O11—S1—O14	113.92 (14)
O7^v^—Rb2—O9	152.12 (7)	O11—S1—O6^ix^	108.97 (14)
O9^vi^—Rb2—O9	90.56 (7)	O14—S1—O6^ix^	111.57 (14)
O2—Rb2—O9	67.50 (7)	O11—S1—O4	109.38 (14)
O13^ii^—Rb2—O9	107.16 (7)	O14—S1—O4	106.03 (14)
O8—Rb2—O9	43.73 (6)	O6^ix^—S1—O4	106.66 (13)
O10^v^—Rb2—O9	158.07 (7)	O12—S2—O9	113.40 (16)
O14^iv^—Rb2—O1	144.45 (6)	O12—S2—O8	114.19 (15)
O7^v^—Rb2—O1	82.45 (6)	O9—S2—O8	111.50 (16)
O9^vi^—Rb2—O1	110.23 (7)	O12—S2—O3	104.03 (14)
O2—Rb2—O1	48.93 (6)	O9—S2—O3	105.97 (15)
O13^ii^—Rb2—O1	78.50 (7)	O8—S2—O3	106.88 (13)
O8—Rb2—O1	56.93 (6)	O13—S3—O7	111.90 (16)
O10^v^—Rb2—O1	120.93 (6)	O13—S3—O10	112.06 (16)
O9—Rb2—O1	79.98 (6)	O7—S3—O10	111.32 (15)
O11—Rb3—O10^ii^	152.16 (7)	O13—S3—O5	108.18 (14)
O11—Rb3—O7^v^	77.41 (7)	O7—S3—O5	108.64 (14)
O10^ii^—Rb3—O7^v^	100.02 (7)	O10—S3—O5	104.36 (14)
O11—Rb3—O14^vii^	138.78 (7)	S2—O3—W1	136.45 (14)
O10^ii^—Rb3—O14^vii^	68.51 (7)	S1—O4—W1	134.71 (14)
O7^v^—Rb3—O14^vii^	110.14 (7)	S3—O5—W1	139.07 (14)
O11—Rb3—O12^i^	75.60 (7)	S1^ix^—O6—W1	130.47 (13)
O10^ii^—Rb3—O12^i^	94.76 (7)		

Symmetry codes: (i) *x*−1/2, −*y*+1/2, *z*−1/2; (ii) *x*+1/2, −*y*+1/2, *z*−1/2; (iii) −*x*+1/2, *y*+1/2, −*z*+3/2; (iv) *x*+1, *y*, *z*; (v) −*x*+1/2, *y*−1/2, −*z*+3/2; (vi) −*x*+1, −*y*, −*z*+2; (vii) −*x*, −*y*, −*z*+1; (viii) *x*−1, *y*, *z*; (ix) −*x*, −*y*, −*z*+2.
